# Early acetaminophen Use and 90-day mortality in ICU patients with ischemic stroke

**DOI:** 10.3389/fphar.2025.1622440

**Published:** 2025-07-24

**Authors:** Zhi-Sheng Piao, Yu-Jia Zhang, Gen Li, Yi Jia, Kun Cheng

**Affiliations:** ^1^Department of Critical Care Medicine, Eastern Hepatobiliary Surgery Hospital Affiliated to Naval Medical University, Shanghai, China; ^2^Department of Emergency, Eastern Hepatobiliary Surgery Hospital Affiliated to Naval Medical University, Shanghai, China; ^3^Department of Obstetrics and Gynecology, Shanghai Changzheng Hospital Affiliated to Naval Medical University, Shanghai, China

**Keywords:** acetaminophen, ischemic stroke, mortality, inverse probability of treatment weighting, intensive care units

## Abstract

**Introduction:**

The impact of acetaminophen on the prognosis of ischemic stroke patients admitted to intensive care units remains unclear. Although acetaminophen is commonly used for fever and pain management, its potential benefits beyond temperature control require further investigation.

**Methods:**

Using the MIMIC-IV database, we retrospectively identified 494 ICU-admitted ischemic stroke patients, of whom 362 (73.28%) received early acetaminophen treatment within 48 h after ICU admission. Patients were stratified based on acetaminophen exposure. Weighted Cox regression was applied after inverse probability of treatment weighting (IPTW) adjustment. Subgroup and sensitivity analyses were performed to assess the consistency of associations.

**Results:**

After IPTW adjustment, early acetaminophen use was associated with reduced 30-day mortality (HR 0.54, 95% CI 0.31–0.94, p = 0.030), and reduced 90-day mortality (HR 0.53, 95% CI 0.32–0.87, p = 0.013). There were no significant differences in in-hospital mortality or hospital length of stay. Subgroup analyses revealed no significant interaction effects, suggesting a consistent association across different clinical strata.

**Discussion:**

Early acetaminophen use may be associated with improved survival outcomes in critically ill ischemic stroke patients. These findings highlight the potential therapeutic value of acetaminophen beyond symptomatic treatment, warranting confirmation through prospective, multicenter randomized controlled trials.

## 1 Introduction

Stroke remains the second leading cause of death worldwide and the third leading cause of death and disability, posing substantial societal and economic challenges (Feigin et al., 2022).Prognostic factors include ischemic injury, reperfusion damage, and hemodynamic instability, such as hypotension, with early inflammation also contributing to worse outcomes (DeLong et al., 2022). Despite advances in reperfusion therapy (Widimsky et al., 2023), antiplatelet and anticoagulation treatments (Mendelson and Prabhakaran, 2021), and lipid-lowering interventions (Amarenco et al., 2020) that have improved patient outcomes, uncertainties persist regarding certain adjunctive therapies. One such unresolved issue is the role of acetaminophen, commonly used for fever control. While temperature management is often pursued, the necessity of aggressive antipyretic therapy remains debated (Fang et al., 2017). Moreover, acetaminophen’s potential organ toxicity raises concerns about its risk-benefit balance in ischemic stroke patients (Ram et al., 2018), and its impact on long-term functional recovery is yet to be definitively established.

Acetaminophen, also known as paracetamol, is a widely utilized antipyretic, analgesic, and anti-inflammatory agent. Its analgesic and thermoregulatory effects are traditionally attributed to the inhibition of cyclooxygenase (COX) enzymes, particularly COX-2 (18). Given its antipyretic properties, acetaminophen is commonly administered to reduce fever in ischemic stroke patients. Beyond its well-known antipyretic effects, acetaminophen has been shown to inhibit peroxidases such as myeloperoxidase, suggesting potential anti-inflammatory properties that could benefit conditions like atherosclerosis and rheumatoid diseases (Marquez and Dunford, 1993). Given the proposed antipyretic and anti-inflammatory mechanisms of acetaminophen, its potential impact on clinical outcomes after ischemic stroke remains an important area for further investigation. In particular, how early acetaminophen use may differentially influence prognosis across patients with varying degrees of temperature elevation, systemic inflammatory responses, and underlying comorbidities warrants systematic exploration. Given the established association between lower body temperature and improved outcomes in patients with acute ischemic stroke, most existing studies have explored the prognostic role of acetaminophen indirectly—primarily through its potential to reduce body temperature. However, current evidence indicates that early administration of acetaminophen is either ineffective (Sulter et al., 2004) or only marginally effective (Koennecke and Leistner, 2001; Dippel D. et al., 2003) in lowering post-stroke temperature. Only a limited number of studies have directly assessed the relationship between acetaminophen use and clinical outcomes in ischemic stroke, and current evidence has yet to clearly establish its potential prognostic benefits (Dippel D. W. et al., 2003; De Ridder et al., 2017; Dippel et al., 2001). Nonetheless, its potential to reduce infarct volume and intracranial pressure following ischemic stroke remains noteworthy and merits attention (Pétrault et al., 2017; Picetti et al., 2014). Particularly in critically ill ischemic stroke patients admitted to the intensive care unit (ICU), the effects of acetaminophen are still poorly understood and require further investigation.

Therefore, we hypothesized that early administration of acetaminophen within 48 h of ICU admission could improve clinical outcomes and reduce mortality in patients with ischemic stroke. To test this hypothesis, we conducted a retrospective cohort study using data from the Medical Information Mart for Intensive Care IV (MIMIC-IV) database (Johnson et al., 2020; Johnson et al., 2023; Goldberger et al., 2000). The primary outcomes included acute care utilization (hospital and ICU length of stay) and mortality outcomes (in-hospital, 30-day, and 90-day mortality). To address potential confounding, we applied inverse probability of treatment weighting (IPTW) to balance baseline covariates, followed by weighted Cox proportional hazards models for time-to-event outcomes and logistic regression for binary endpoints.

## 2 Methods

### 2.1 Data source

This retrospective cohort study utilized data from the MIMIC-IV database (version 3.1), which includes electronic health records of ICU patients admitted to Beth Israel Deaconess Medical Center, United States. The MIMIC-IV database itself was approved by the Institutional Review Board (IRB) of Beth Israel Deaconess Medical Center (approval number: 2001P-001699/14). One of the authors (Zhisheng Piao) has completed the Collaborative Institutional Training Initiative (CITI) examination (Certification number: 54,170,610) and is authorized to access the database. As the MIMIC-IV database contains de-identified patient data, further ethical approval was not required for this study.

### 2.2 Participants

**FIGURE 1:**
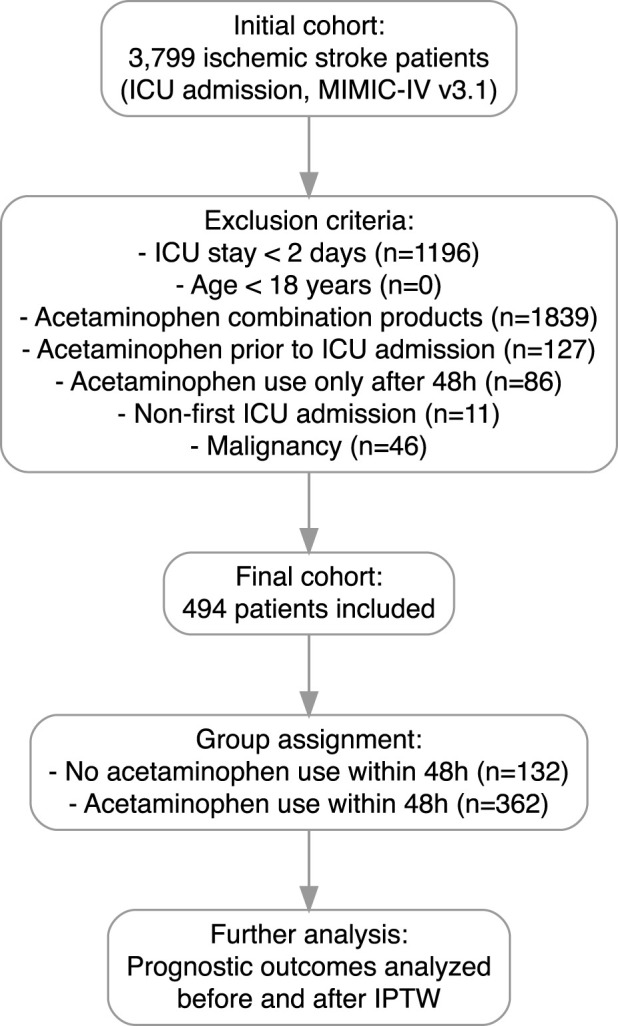
Flowchart of patient selection. A total of 3,799 patients diagnosed with ischemic stroke were initially screened from the MIMIC-IV v3.1 database. Patients were excluded if they had an ICU stay of less than 2 days, were younger than 18 years, used acetaminophen combination products during hospitalization, received acetaminophen prior to ICU admission, initiated acetaminophen use only after 48 h of ICU admission, had a non-first ICU admission, or had a documented malignancy. After applying these criteria, 494 patients were included in the final cohort. Patients were then categorized into two groups based on acetaminophen exposure within the first 48 h of ICU admission.

Patients diagnosed with ischemic stroke and admitted to the ICU were identified from the MIMIC-IV version 3.1 database, based on diagnoses from the hospital diagnoses icd table. A total of 3,799 patients were initially screened. Patients were excluded if they had an ICU stay of less than 2 days, were younger than 18 years, used acetaminophen combination products during hospitalization, received acetaminophen prior to ICU admission, initiated acetaminophen use only after 48 h of ICU admission, had a non-first ICU admission, or had a documented malignancy. After applying these criteria, 494 patients were included in the final cohort. Patients were subsequently categorized into two groups based on acetaminophen exposure within the first 48 h of ICU admission: those without acetaminophen use (n = 132) and those with acetaminophen use (n = 362). Prognostic outcomes were analyzed before and after adjustment by IPTW to minimize confounding. The flowchart of patient selection is shown in Figure 1.

### 2.3 Covariates

Baseline covariates were extracted, including demographic characteristics (age, sex, race, weight), severity of illness scores (Sequential Organ Failure Assessment [SOFA] score and minimum Glasgow Coma Scale [GCS] score), laboratory measurements (C-reactive protein [CRP], hematocrit, platelet count, maximum white blood cell [WBC] count, maximum glucose level, mean anion gap, mean bicarbonate level, maximum blood urea nitrogen [BUN], mean calcium level, mean chloride level, maximum creatinine level, mean sodium level, maximum potassium level, maximum prothrombin time [PT], maximum partial thromboplastin time [PTT], maximum alanine aminotransferase [ALT], maximum alkaline phosphatase [ALP], maximum aspartate aminotransferase [AST], and maximum total bilirubin level), and vital signs (mean heart rate, mean arterial pressure [MAP], mean respiratory rate, maximum temperature, minimum oxygen saturation ${[\text{SpO}}_{2}$], and total urine output during ICU stay). In addition, comorbidities (hypertension, atrial fibrillation, myocardial infarction, congestive heart failure, chronic pulmonary disease, rheumatic disease, peptic ulcer disease, diabetes mellitus, paraplegia, renal disease, and liver disease) and treatments (use of statins and antiplatelet drugs) were recorded.

For variables with multiple daily measurements, we selected values representing the most severe status on the first ICU day based on clinical relevance and extracted mean values where appropriate to reflect the overall condition. Missing data were minimal (<30% for all variables). Multiple imputation using chained equations was performed to address missingness, employing the random forest method with five imputations, ten iterations, and 100 trees via the “mice” package in R software (version 4.4.2).

### 2.4 Statistical analysis

Continuous variables were described as mean ± standard deviation (SD) if normally distributed and as median (in-terquartile range, IQR) if not normally distributed. Between-group comparisons were conducted using the independent t-test for normally distributed variables and the Mann-Whitney U test for non-normally distributed variables. Categorical variables were expressed as n (%) and compared between groups using the chi-square test.

To control for potential confounding, IPTW based on propensity scores was employed. Propensity scores were estimated using logistic regression incorporating all baseline covariates, including demographic characteristics, severity of illness scores, laboratory measurements, vital signs, comorbidities, and treatments. We included all clinically relevant covariates available from the MIMIC-IV database to adjust for baseline differences between groups as comprehensively as possible. The MatchIt package (version 4.7.1) in R was used to calculate propensity scores, and the WeightIt (version 1.4.0) package was used to derive the IPTW weights. After weighting, stabilized weights were calculated, and to minimize the influence of extreme weights and improve estimate stability, we applied truncation at the 1st and 95th percentiles of the weight distribution. Covariate balance before and after weighting was assessed by calculating standardized mean differences (SMDs), with an absolute SMD <0.2 considered indicative of acceptable balanceCovariate balance was assessed using SMDs, with an absolute SMD <0.2 considered indicative of acceptable balance, and values below 0.1 interpreted as indicative of excellent balance (Cohen, 2013; Chesnaye et al., 2021). The detailed SMD values are presented in Table 1.

**TABLE 1:** Baseline characteristics before and after IPTW adjustment in patients with and without acetaminophen use.

Variables	Before weighting				After weighting			
	Non-acetaminophen (n = 132)	Acetaminophen (n = 362)	p-value	SMD	Non-acetaminophen (n = 132)	Acetaminophen (n = 362)	p-value	SMD
age (mean (SD))	71.88 (14.96)	69.87 (15.34)	0.195	0.133	71.75 (14.41)	70.68 (15.24)	0.517	0.072
gender (%)								
Male	69 (52.3)	171 (47.2)	0.374	0.101	59.3 (53.1)	169.5 (49.0)	0.478	0.083
female	63 (47.7)	191 (52.8)			52.3 (46.9)	176.3 (51.0)		
race (%)								
White	80 (60.6)	207 (57.2)	0.562	0.07	67.5 (60.5)	202.0 (58.4)	0.722	0.042
Other	52 (39.4)	155 (42.8)			44.1 (39.5)	143.7 (41.6)		
weight (mean (SD))	79.42 (22.28)	80.91 (23.92)	0.534	0.064	79.29 (21.67)	80.42 (23.63)	0.649	0.05
severity of illness								
sofa (mean (SD))	4.20 (3.51)	2.58 (2.10)	<0.001	0.561	3.20 (2.74)	2.77 (2.16)	0.09	0.173
gcs (mean (SD))	12.60 (3.14)	13.16 (2.50)	0.039	0.199	12.83 (2.81)	13.11 (2.49)	0.359	0.105
laboratory measurements								
crp (mean (SD))	49.70 (65.13)	32.06 (57.76)	0.004	0.287	37.06 (57.77)	34.97 (61.12)	0.738	0.035
hematocrit (mean (SD))	37.73 (6.49)	37.80 (6.04)	0.909	0.011	38.12 (5.62)	37.86 (6.16)	0.667	0.044
platelet (mean (SD))	232.98 (125.31)	224.54 (75.09)	0.363	0.082	221.46 (100.11)	223.68 (77.74)	0.816	0.025
wbc (mean (SD))	12.50 (5.64)	11.00 (4.63)	0.003	0.291	11.27 (4.72)	11.08 (4.72)	0.699	0.041
glucose (mean (SD))	184.71 (85.99)	151.24 (60.24)	<0.001	0.451	166.45 (72.54)	155.99 (67.24)	0.179	0.15
aniongap (mean (SD))	14.82 (3.82)	14.08 (2.80)	0.019	0.223	14.41 (3.38)	14.15 (2.79)	0.475	0.083
bicarbonate (mean (SD))	22.55 (3.71)	23.19 (3.04)	0.05	0.19	23.09 (3.27)	23.17 (3.03)	0.809	0.027
bun (mean (SD))	25.25 (15.67)	20.34 (12.65)	<0.001	0.345	21.73 (13.54)	21.04 (13.20)	0.633	0.051
calcium (mean (SD))	8.81 (0.71)	8.79 (0.57)	0.842	0.019	8.86 (0.64)	8.81 (0.57)	0.427	0.091
chloride (mean (SD))	103.59 (5.68)	104.03 (4.25)	0.347	0.089	103.81 (4.68)	103.99 (4.38)	0.698	0.041
creatinine (mean (SD))	1.32 (0.73)	1.12 (0.74)	0.009	0.269	1.19 (0.62)	1.15 (0.78)	0.585	0.055
sodium (mean (SD))	139.45 (4.20)	139.84 (3.45)	0.293	0.102	139.78 (3.64)	139.82 (3.59)	0.899	0.014
potassium (mean (SD))	4.18 (0.65)	4.11 (0.52)	0.203	0.123	4.13 (0.63)	4.11 (0.52)	0.852	0.022
pt (mean (SD))	16.08 (8.69)	14.15 (4.68)	0.002	0.277	14.59 (6.53)	14.30 (4.69)	0.616	0.05
ptt (mean (SD))	47.52 (34.94)	42.47 (28.02)	0.099	0.159	45.39 (32.13)	42.58 (28.26)	0.439	0.093
alt (mean (SD))	132.14 (474.12)	31.69 (81.63)	<0.001	0.295	68.43 (285.24)	36.09 (103.73)	0.061	0.151
alp (mean (SD))	96.30 (56.61)	85.75 (41.22)	0.024	0.213	87.72 (43.95)	86.64 (42.19)	0.794	0.025
ast (mean (SD))	256.64 (822.29)	48.85 (159.47)	<0.001	0.351	112.37 (496.76)	54.69 (175.93)	0.044	0.155
bilirubin (mean (SD))	0.83 (1.10)	0.66 (0.54)	0.022	0.197	0.71 (0.73)	0.67 (0.54)	0.507	0.059
vital signs								
heart rate (mean (SD))	83.50 (17.19)	77.96 (12.89)	<0.001	0.364	79.59 (16.31)	78.82 (13.13)	0.65	0.052
mbp (mean (SD))	89.09 (13.27)	91.62 (12.52)	0.051	0.197	91.29 (12.85)	91.39 (12.53)	0.945	0.008
resp rate (mean (SD))	19.57 (4.27)	18.63 (2.80)	0.005	0.26	19.09 (3.53)	18.75 (2.90)	0.339	0.104
temperature (mean (SD))	37.33 (0.56)	37.34 (0.52)	0.818	0.023	37.30 (0.49)	37.33 (0.52)	0.589	0.057
spo2 (mean (SD))	91.21 (5.57)	92.67 (3.50)	0.001	0.314	92.04 (4.70)	92.44 (3.63)	0.387	0.095
urineoutput (mean (SD))	1410.89 (912.94)	1592.75 (953.34)	0.058	0.195	1533.90 (861.19)	1559.56 (961.84)	0.796	0.028
comorbidities								
hypertension (%)								
No	123 (93.2)	322 (89.0)	0.222	0.149	103.1 (92.3)	311.3 (90.0)	0.513	0.081
Yes	9 (6.8)	40 (11.0)			8.6 (7.7)	34.5 (10.0)		
atrial fibrillation (%)								
No	84 (63.6)	225 (62.2)	0.845	0.031	71.8 (64.3)	214.5 (62.0)	0.684	0.047
Yes	48 (36.4)	137 (37.8)			39.9 (35.7)	131.3 (38.0)		
myocardial infarct (%)								
No	109 (82.6)	309 (85.4)	0.537	0.076	96.0 (86.0)	296.5 (85.8)	0.947	0.008
Yes	23 (17.4)	53 (14.6)			15.6 (14.0)	49.2 (14.2)		
congestive heart failure (%)								
No	84 (63.6)	279 (77.1)	0.004	0.297	81.3 (72.8)	259.4 (75.0)	0.644	0.05
Yes	48 (36.4)	83 (22.9)			30.4 (27.2)	86.4 (25.0)		
chronic pulmonary disease (%)								
No	115 (87.1)	310 (85.6)	0.783	0.043	95.9 (85.9)	296.2 (85.7)	0.945	0.008
Yes	17 (12.9)	52 (14.4)			15.7 (14.1)	49.6 (14.3)		
rheumatic disease (%)								
No	126 (95.5)	352 (97.2)	0.482	0.095	106.8 (95.7)	334.9 (96.9)	0.58	0.063
Yes	6 (4.5)	10 (2.8)			4.8 (4.3)	10.8 (3.1)		
peptic ulcer disease (%)								
No	130 (98.5)	358 (98.9)	1.000	0.036	110.9 (99.3)	342.3 (99.0)	0.658	0.035
Yes	2 (1.5)	4 (1.1)			0.8 (0.7)	3.4 (1.0)		
diabetes (%)								
No	82 (62.1)	249 (68.8)	0.199	0.14	71.6 (64.1)	231.1 (66.8)	0.623	0.057
Yes	50 (37.9)	113 (31.2)			40.1 (35.9)	114.7 (33.2)		
paraplegia (%)								
No	62 (47.0)	150 (41.4)	0.319	0.112	46.1 (41.3)	144.1 (41.7)	0.948	0.008
Yes	70 (53.0)	212 (58.6)			65.5 (58.7)	201.7 (58.3)		
renal disease (%)								
No	109 (82.6)	308 (85.1)	0.589	0.068	91.8 (82.2)	288.8 (83.5)	0.763	0.035
Yes	23 (17.4)	54 (14.9)			19.9 (17.8)	57.0 (16.5)		
liver disease (%)								
No	124 (93.9)	355 (98.1)	0.039	0.212	107.9 (96.6)	336.7 (97.4)	0.646	0.044
Yes	8 (6.1)	7 (1.9)			3.8 (3.4)	9.1 (2.6)		
Treatments								
statins (%)								
No	68 (51.5)	105 (29.0)	<0.001	0.472	39.9 (35.7)	112.9 (32.6)	0.556	0.065
Yes	64 (48.5)	257 (71.0)			71.8 (64.3)	232.9 (67.4)		
antiplatelet drugs (%)								
No	69 (52.3)	115 (31.8)	<0.001	0.425	45.1 (40.4)	123.1 (35.6)	0.381	0.099
Yes	63 (47.7)	247 (68.2)			66.5 (59.6)	222.7 (64.4)		

Data are presented as mean (SD) for continuous variables and number (percentage) for categorical variables. SMD, standardized mean difference; IPTW, inverse probability of treatment weighting. A p-value <0.05 or an SMD >0.1 indicates a meaningful imbalance between groups.

The associations between acetaminophen exposure and clinical outcomes were analyzed both before and after IPTW adjustment. Linear regression models were used to evaluate continuous outcomes, including hospital length of stay and ICU length of stay. Logistic regression models were employed to assess the relationship between acetaminophen exposure and in-hospital mortality. Cox proportional hazards models were applied to examine the associations between acetaminophen use and 30-day as well as 90-day mortality outcomes.

Before IPTW adjustment, multivariable models were constructed with progressive adjustment for covariates (Model 1, Model 2, and Model 3). After IPTW adjustment, given that the covariate balance was achieved SMDs <0.1 and P > 0.05), no further covariate adjustment was performed in the weighted analyses. Kaplan-Meier survival curves were generated using the survival, survminer, and ggplot2 packages in R.

To test the robustness of the findings, subgroup analyses were conducted both before and after IPTW adjustment. Stratified analyses were performed across the following predefined subgroups: age (≥65 vs <65 years), sex (male vs female), race (White vs other), CRP level (≥8 mg/L vs <8 mg/L), maximum WBC, ≥10.2 $\times 10^{9}$ /L vs <10.2 $\times 10^{9}$ /L), maximum temperature (≥37.3°C vs <37.3°C), presence of hypertension, atrial fibrillation, myocardial infarction, congestive heart failure, chronic pulmonary disease, renal disease, liver disease, and use of statins or antiplatelet agents.The interaction between acetaminophen use and each subgroup variable was tested, and the results were visualized using forest plots created with the forestplot package in R.

Data processing and analysis were performed using R (version 4.4.2). Statistical significance was defined as p < 0.05.

## 3 Results

### 3.1 Baseline characteristics before and after IPTW adjustment

As shown in Figure 1, after applying the inclusion and exclusion criteria, a total of 494 patients with ischemic stroke admitted to the ICU were included in the study cohort, comprising 362 patients who received early acetaminophen use and 132 patients who did not. Baseline characteristics, including demographics, laboratory results, comorbidities, vital signs, and treatments, were systematically compared between the two groups (Table 1).

Before IPTW adjustment, significant differences were observed between groups. Patients who did not receive acetaminophen had higher severity of illness, as indicated by higher SOFA scores (4.20 vs 2.58, p < 0.001; SMD = 0.561) and lower minimum GCS scores (12.60 vs 13.16, p = 0.039; SMD = 0.199), suggesting greater initial disease severity. Markers of inflammation, including CRP (49.70 vs 32.06 mg/L, p = 0.004; SMD = 0.287) and maximum WBC (12.50 vs 11.00 × $10^{9}$ /L, p = 0.003; SMD = 0.291), were significantly higher in the non-acetaminophen group, indicating a stronger inflammatory response.

Additionally, patients without early acetaminophen use exhibited higher glucose levels, BUN, creatinine, PT, liver enzymes (ALT and AST), and bilirubin, along with faster heart rate and respiratory rate and lower minimum ${\text{SpO}}_{2}$ . Comorbidities such as congestive heart failure (36.4% vs 22.9%, p = 0.004; SMD = 0.297) and liver disease (6.1% vs 1.9%, p = 0.039; SMD = 0.212) were more prevalent among patients not receiving acetaminophen. In terms of treatments, fewer patients in the non-acetaminophen group received statins (48.5% vs 71.0%, p < 0.001; SMD = 0.472) or antiplatelet therapy (47.7% vs 68.2%, p < 0.001; SMD = 0.425).

These findings suggest that prior to weighting, patients who did not receive early acetaminophen were generally sicker, exhibited more pronounced inflammatory responses, and had a higher burden of comorbidities compared with those who received acetaminophen.

Prior to weighting, substantial imbalances were observed between the acetaminophen and non-acetaminophen groups. After applying IPTW using the propensity score, the baseline covariates were effectively balanced, with all SMDs reduced to less than 0.2 and p-values exceeding 0.05. These results suggest that the two groups were comparable with respect to baseline characteristics following weighting.

### 3.2 Association between acetaminophen use and clinical outcomes before IPTW adjustment

We further examined the association between acetaminophen use and clinical outcomes including hospital length of stay, ICU length of stay, in-hospital mortality, and short-term (30-day and 90-day) mortality. As shown in Table 2, there were no significant differences in hospital or ICU stay between the acetaminophen and non-acetaminophen groups across all models. In contrast, the unadjusted analysis showed that acetaminophen use was significantly associated with a reduced risk of in-hospital mortality (OR 0.36, 95% CI 0.21–0.62; p < 0.001). However, after full adjustment in Model 3, the association was attenuated and no longer statistically significant (OR 0.54, 95% CI 0.25–1.21; p = 0.132).

**TABLE 2:** Association between acetaminophen use and clinical outcomes before IPTW adjustment.

Outcome	Non-Acetaminophen (n = 132)	Acetaminophen (n = 362)	Unadjusted (Est. [95% CI], p)	Model 1(Est. [95% CI], p)	Model 2(Est. [95% CI], p)	Model 3(Est. [95% CI], p)
Length of hospital stay (median [IQR], days)	5.57 [3.71, 9.61]	6.07 [4.01, 10.57]	−0.38 (−2.08, 1.32), p = 0.661	−0.45 (−2.14, 1.24), p = 0.601	0.23 (−1.43, 1.89), p = 0.787	0.69 (−1.12, 2.5), p = 0.455
In-hospital mortality (%)	30 (22.7)	35 (9.7)	OR 0.36 (0.21, 0.62), p < 0.001	OR 0.35 (0.20, 0.61), p < 0.001	OR 0.34 (0.19, 0.60), p < 0.001	OR 0.54 (0.25, 1.21), p = 0.132
Length of ICU stay (median [IQR], days)	3.49 [2.84, 5.21]	3.82 [2.79, 5.39]	0.45 (−0.43, 1.33), p = 0.312	0.35 (−0.52, 1.22), p = 0.424	0.64 (−0.22, 1.50), p = 0.142	0.77 (−0.15, 1.69), p = 0.100
30-day mortality (%)	40 (30.3)	47 (13.0)	HR 0.37 (0.25, 0.57), p < 0.001	HR 0.38 (0.25, 0.58), p < 0.001	HR 0.38 (0.25, 0.59), p < 0.001	HR 0.50 (0.30, 0.85), p = 0.009
90-day mortality (%)	51 (38.6)	63 (17.4)	HR 0.38 (0.26, 0.55), p < 0.001	HR 0.39 (0.27, 0.57), p < 0.001	HR 0.41 (0.28, 0.60), p < 0.001	HR 0.52 (0.33, 0.81), p = 0.004

Abbreviations: IQR, interquartile range; OR, odds ratio; HR, hazard ratio; CI, confidence interval.

Model 1: adjusted for age, gender, race, and weight at admission.

Model 2: Model 1 + comorbidities (hypertension, atrial fibrillation, myocardial infarction, congestive heart failure, chronic pulmonary disease, renal disease, liver disease).

Model 3: Model 2 + clinical scores (APACHE III, SOFA, GCS), lab values (CRP, hematocrit, platelet; WBC, glucose, creatinine), vital signs (heart rate, MBP, respiratory rate, temperature, ${\text{SpO}}_{2}$ ), and treatments (statins, antiplatelet drugs).

**FIGURE 2:**
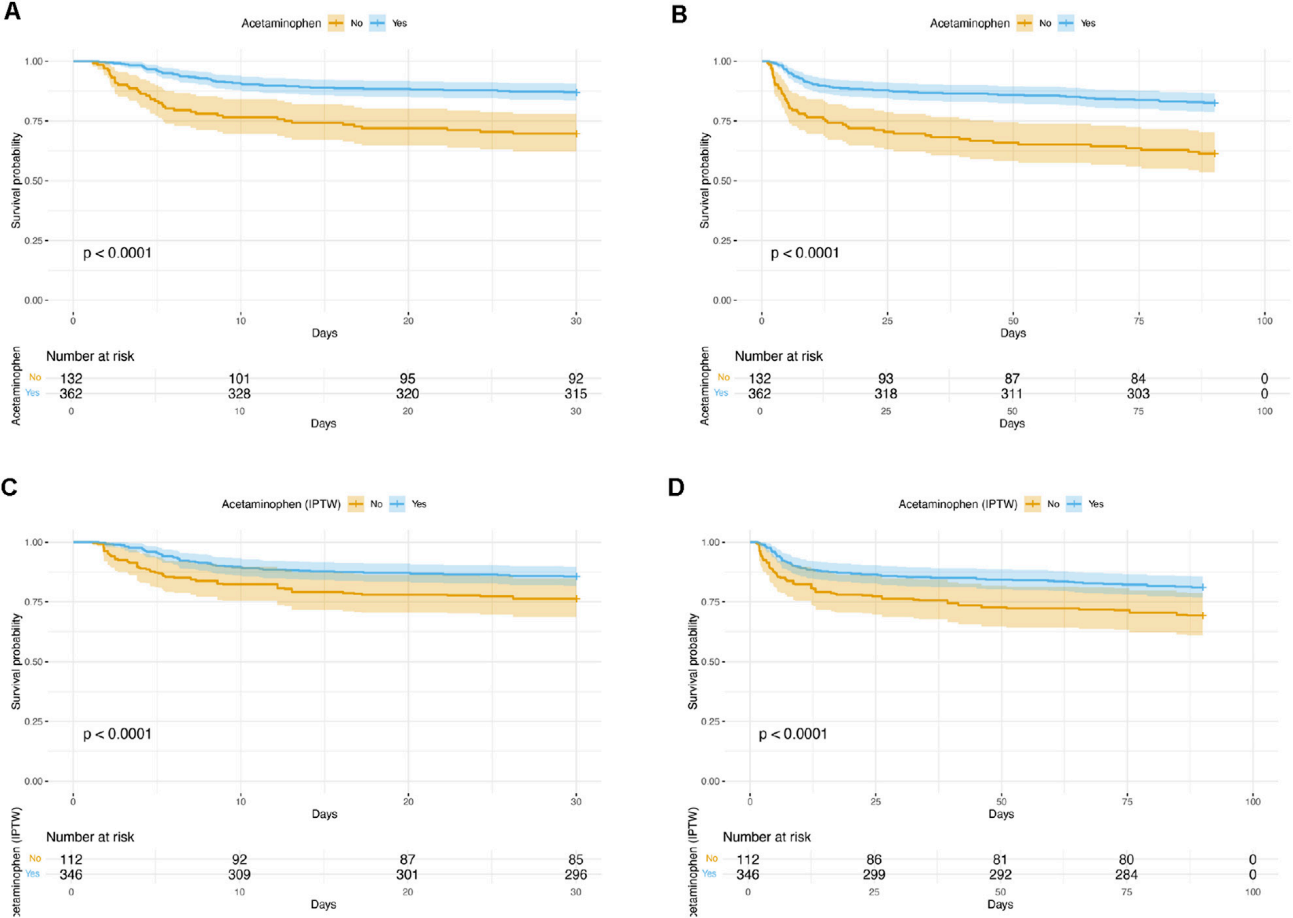
Kaplan–Meier Survival Curves Comparing acetaminophen Users and Non-Users. **(A)** 30-day survival before IPTW adjustment; **(B)** 90-day survival before IPTW adjustment; **(C)** 30-day survival after IPTW adjustment; **(D)** 90-day survival after IPTW adjustment. In all analyses, patients who received acetaminophen had significantly higher survival rates compared to non-users (log-rank test, P < 0.0001 for all comparisons). IPTW = inverse probability of treatment weighting.

In terms of 30-day and 90-day mortality, acetaminophen use was consistently associated with significantly lower mortality risks both before and after adjustment. In Model 3, acetaminophen use was associated with a 50% reduction in 30-day mortality (HR 0.50, 95% CI 0.30–0.85; p = 0.009) and a 48% reduction in 90-day mortality (HR 0.52, 95% CI 0.33–0.81; p = 0.004), indicating a robust association with improved short-term survival. The Kaplan–Meier survival curves demonstrated that patients receiving acetaminophen had significantly higher survival rates compared to non-users at both 30 days (Figure 2A) and 90 days (Figure 2B) before IPTW adjustment (all P < 0.0001).

### 3.3 Association between acetaminophen use and clinical outcomes after IPTW adjustment

After IPTW adjustment, the association between acetaminophen use and clinical outcomes was further evaluated (Table 3). The weighted analysis showed that the acetaminophen group had a slightly longer median hospital stay compared to the non-acetaminophen group; however, the difference did not reach statistical significance (1.25 days, 95% CI: 0.17 to 2.66; p = 0.084). There was no significant difference in in-hospital mortality between the two groups (OR 0.95, 95% CI: 0.88 to 1.02; p = 0.182). Notably, acetaminophen use was associated with a significantly longer ICU stay (0.87 days, 95% CI: 0.21 to 1.54; p = 0.010). Importantly, acetaminophen use remained significantly associated with a lower risk of 30-day mortality (HR 0.54, 95% CI: 0.31 to 0.94; p = 0.030) and 90-day mortality (HR 0.53, 95% CI: 0.32 to 0.87; p = 0.013) after IPTW adjustment. The IPTW-weighted Kaplan–Meier survival curves confirmed these findings, showing significantly better survival in the acetaminophen group at both 30 days (Figure 2C) and 90 days (Figure 2D) after IPTW adjustment (all P < 0.0001).

**TABLE 3:** Association between acetaminophen use and clinical outcomes after IPTW adjustment.

Outcome	Non-Acetaminophen (n = 111.65)	Acetaminophen (n = 345.78)	Weighted effect (Est. [95% CI], p)
Length of hospital stay (median [IQR], days)	4.92 [3.40, 8.06]	6.04 [4.01, 10.63])	1.25 (−0.17, 2.66), p = 0.084
In-hospital mortality (%)	17.6 (15.7)	37.0 (10.7)	OR 0.95 (0.88, 1.02), p = 0.182
Length of ICU stay (median [IQR], days)	3.22 [2.67, 4.74]	3.79 [2.76, 5.30]	0.87 (0.21, 1.54), p = 0.010
30-day mortality (%)	26.4 (23.7)	49.7 (14.4)	HR 0.54 (0.31, 0.94), p = 0.030
90-day mortality (%)	34.3 (30.7)	65.4 (18.9)	HR 0.53 (0.32, 0.87), p = 0.013

Abbreviations: IQR, interquartile range; OR, odds ratio; HR, hazard ratio; CI, confidence interval. Effect estimates are obtained after inverse probability of treatment weighting (IPTW).

### 3.4 Sensitivity and subgroup analyses of acetaminophen and mortality

**FIGURE 3:**
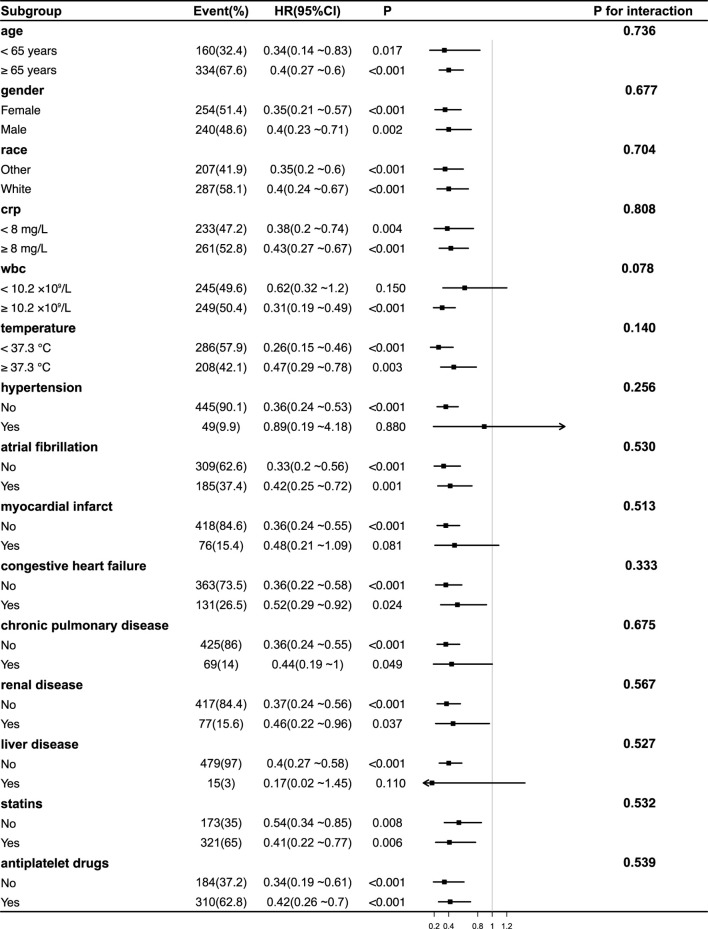
Subgroup Analyses of the Association Between acetaminophen Use and 90-Day Mortality Before IPTW Adjustment. Forest plot showing hazard ratios (HRs) and 95% confidence intervals (CIs) for the association between acetaminophen use and 90-day mortality across various patient subgroups before inverse probability of treatment weighting (IPTW) adjustment. No significant interaction effects were observed across subgroups (all P for interaction >0.05), indicating consistent benefits of acetaminophen use. Notably, the protective effect of acetaminophen was consistent across different inflammatory statuses, including CRP levels, WBC counts, and body temperature groups. Abbreviations: HR, hazard ratio; CI, confidence interval; CRP, C-reactive protein; WBC, white blood cell; IPTW, inverse probability of treatment weighting.

**FIGURE 4:**
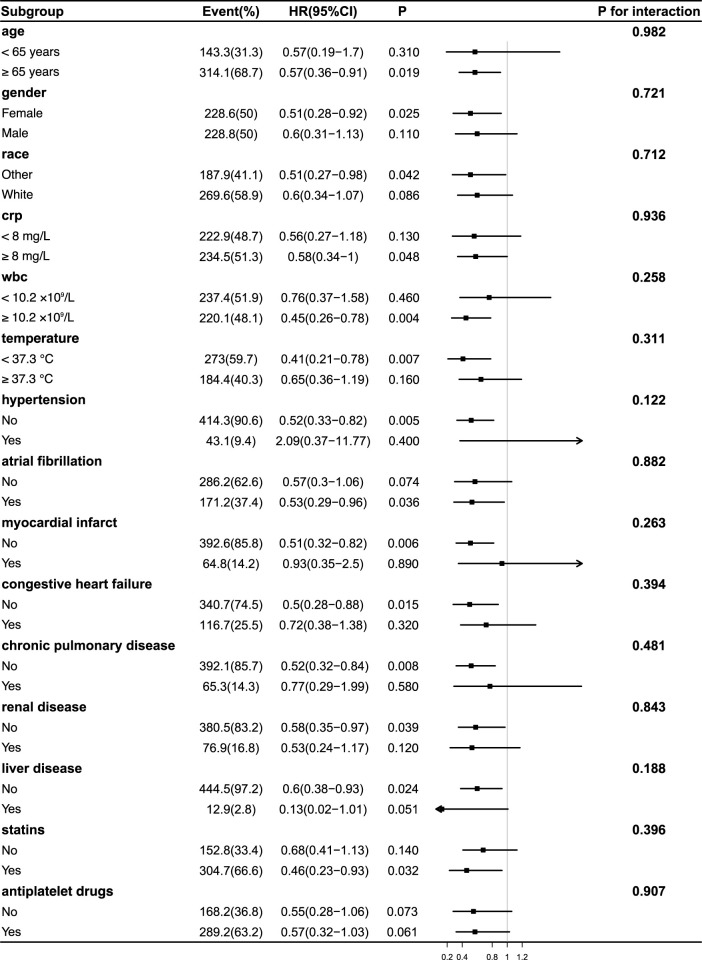
Subgroup Analyses of the Association Between acetaminophen Use and 90-Day Mortality After IPTW Adjustment. Forest plot illustrating hazard ratios (HRs) and 95% confidence intervals (CIs) for 90-day mortality associated with acetaminophen use in various subgroups following inverse probability of treatment weighting (IPTW). No significant interaction was observed between treatment effect and any subgroup (all P for interaction >0.05), indicating a consistent survival benefit across populations. The effect remained stable in patients with differing levels of inflammation, including CRP, WBC, and body temperature subgroups. Abbreviations: HR, hazard ratio; CI, confidence interval; CRP, C-reactive protein; WBC, white blood cell; IPTW, inverse probability of treatment weighting.

Subgroup analyses were conducted to assess the consistency of the association between acetaminophen use and 90-day mortality across clinical and demographic characteristics (Figures 3, 4). In both unweighted and IPTW-weighted models, the protective association remained stable across all examined subgroups, with no significant interaction effects observed (all P for interaction > 0.05). These subgroups included age (<65 vs. ≥65 years), sex, race, and the presence of major comorbidities such as cardiovascular, hepatic, and renal diseases. We also examined the influence of concurrent treatments, including antiplatelet and lipid-lowering therapies, which may reflect differences in overall clinical management.

Notably, the potential survival benefit of acetaminophen was consistent across subgroups stratified by inflammatory status, including CRP (<8 vs. ≥8 mg/L), WBC counts (<10.2 vs. ≥10.2 × $10^{9}$ /L), and maximum body temperature (<37.3°C vs ≥37.3°C), suggesting that its effect may be independent of baseline systemic inflammation. Taken together, these results suggest that the association between acetaminophen use and improved survival is robust across various subgroups within the context of the current analysis, despite baseline heterogeneity.

## 4 Discussion

In this study, acetaminophen use was associated with a lower risk of 30-day and 90-day mortality among critically ill patients, even after adjustment with IPTW. Kaplan-Meier analyses consistently showed higher survival rates in the acetaminophen group before and after weighting (all p < 0.0001). Subgroup and sensitivity analyses demonstrated no significant interaction effects, suggesting that the observed survival benefit was consistent across different age groups, sex, race, and levels of inflammatory response. These findings suggest a potential association between acetaminophen use and improved survival, although causality cannot be confirmed.

Standard treatments for ischemic stroke primarily include mechanical thrombectomy, thrombolysis, and antiplatelet therapy. In clinical practice, acetaminophen is mainly used in ischemic stroke patients for both fever and pain management. Studies have shown that each 1°C increase in body temperature within the first 12 h after stroke can double the risk of poor functional outcomes (Reith et al., 1996). Current guidelines recommend antipyretic treatment for elevated temperatures: the American Stroke Association suggests intervention when the temperature exceeds 38°C (Adams et al., 2007), while the Canadian Guidelines propose a threshold of 37.5°C (Casaubon et al., 2016). Early administration of acetaminophen has been shown to effectively reduce body temperature within the first 24 h following ischemic stroke; however, it does not significantly influence temperature control beyond this period (Dippel D. W. et al., 2003; Dippel et al., 2001). Moreover, existing studies have not demonstrated a significant effect of acetaminophen-mediated fever reduction on mortality (Kasner et al., 2002).

Beyond temperature control, acetaminophen has been reported to exert potential protective effects in other populations. Although concerns exist regarding drug toxicity—including risks of acute kidney injury (AKI), liver damage, and cardiovascular events (Yarema et al., 2021; Karvellas et al., 2017) — these adverse outcomes are largely associated with overdose, and routine use does not appear to elevate cardiovascular risk (Rao et al., 2023). Some studies suggest that acetaminophen may reduce the risk of AKI in postoperative cardiac surgery patients (Xiong et al., 2023), though evidence in septic patients remains inconsistent (Hiragi et al., 2018). Similarly, findings on its impact on patient prognosis are mixed: while acetaminophen does not significantly affect all-cause mortality in general febrile patients after adjusting for confounders (Rao et al., 2023), it may reduce mortality in septic critically ill patients (Janz et al., 2013). Conversely, its immunosuppressive properties could negate benefits in infections caused by Gram-negative bacteria (Mohr et al., 2012). In ischemic stroke patients, acetaminophen has not been associated with significant improvements in functional outcomes or serious adverse event rates when compared to placebo (de Ridder et al., 2015). Although the relationship between elevated body temperature and worse prognosis is well-established (Karaszewski et al., 2012; Wang et al., 2000), temperature-lowering interventions have not consistently been shown to reduce mortality.

Given that no significant intergroup differences in body temperature were observed after IPTW adjustment, the observed survival benefit is unlikely to be solely attributed to temperature regulation. This raises the possibility that acetaminophen may exert additional protective effects in critically ill ischemic stroke patients. Potential mechanisms may involve its antioxidant properties, modulation of inflammatory responses, or attenuation of secondary neuronal injury. However, further studies are needed to elucidate the precise biological pathways underlying these findings. Previous studies have shown that the temperature-lowering effect of acetaminophen diminishes after 24 h, making it difficult to attribute changes in long-term outcomes solely to temperature regulation (Kasner et al., 2002). Acetaminophen is now understood to inhibit COX-1 and COX-2 through the peroxidase activity of these enzymes. Unlike non-selective NSAIDs and selective COX-2 inhibitors, acetaminophen, despite lacking classical NSAID-mediated anti-inflammatory effects, also inhibits other peroxidases, including myeloperoxidase (MPO) (Graham et al., 2013). MPO, a pro-inflammatory enzyme secreted in large quantities by activated myeloid cells following stroke, plays a critical role in the pathogenesis of both hemorrhagic and ischemic strokes, contributing to blood-brain barrier (BBB) disruption and subsequent brain injury (Wang et al., 2022). Following acute cerebral ischemia, BBB damage facilitates substantial neutrophil infiltration into the central nervous system, accompanied by increased MPO production (Gorudko et al., 2017). Animal studies have demonstrated that MPO inhibition significantly improves post-stroke neurological outcomes, with a reported 60% increase in Hsp70+/NeuN + neuronal survival (Kim et al., 2019). The ability of acetaminophen to inhibit MPO activity and reduce the production of potent oxidants such as hypobromous acid (HOBr) and hypochlorous acid (HOCl) may partially explain its potential protective effects in ischemic stroke patients (Morgan et al., 2011).

Recent studies have suggested that the potential effects of acetaminophen may extend beyond its classical cyclooxygenase inhibition pathway and involve multiple mechanisms of action. One such mechanism is through the formation of the bioactive metabolite AM404 within the central nervous system. Following deacetylation to p-aminophenol, acetaminophen is conjugated with arachidonic acid in the brain and spinal cord to form AM404—a potent TRPV1 agonist known to influence endocannabinoid signaling and exert neuroprotective effects (Mallet et al., 2023; Högestätt et al., 2005). In a rodent model of cerebral ischemia-reperfusion injury, Sherif S. Abdel Mageed et al. demonstrated that acetaminophen exerted neuroprotective effects by modulating central AM404 levels (Mageed et al., 2022). Additionally, *in vitro* studies have shown that acetaminophen attenuates oxidative stress by suppressing the release of pro-inflammatory cytokines such as TNF- α , interleukin-1, and macrophage inflammatory protein-1 α (Tripathy and Grammas, 2009). Consistent findings by Aya Yassin Labib further support acetaminophen’s multi-target neuroprotective properties involving oxidative stress reduction, anti-inflammatory activity, modulation of apoptotic pathways, and cannabinoid receptor regulation. Moreover, acetaminophen may alleviate postictal vasoconstriction, cerebral hypoperfusion, and subsequent tissue hypoxia—events commonly associated with post-stroke seizures or epilepsy—via its traditional COX-mediated effects (Farre et al., 2016). Seizures and status epilepticus are recognized complications of acute ischemic stroke, occurring in approximately 4% of patients and potentially leading to long-term epilepsy (Vercueil, 2007; Farrell et al., 2017). These complications may further exacerbate cerebral hypoxia and metabolic stress, suggesting that acetaminophen’s modulatory effects on both inflammation and cerebral perfusion may have broader implications in the post-stroke setting.

In our current subgroup analyses presented in Figures 3, 4, no significant interaction effects were observed between acetaminophen treatment and the stratified variables. This includes stratifications based on WBC levels, body temperature, and CRP levels. Additionally, comorbidities such as cardiac disease, renal disease, and liver disease did not significantly modify the effect of acetaminophen intervention. These findings suggest that the potential benefits of acetaminophen in ischemic stroke patients may extend across different inflammatory statuses and comorbidity profiles. Notably, these observations provide additional support for previous research (Rao et al., 2023), which indicated that acetaminophen use does not adversely impact the prognosis in patients with chronic comorbidities.

Our findings suggesting a potential benefit of early acetaminophen use on the outcomes of ICU-admitted ischemic stroke patients are encouraging. In our study, we initially conducted multivariable Cox regression analyses in the original cohort to adjust for potential confounders, and subsequently applied IPTW to further balance baseline characteristics between groups. After weighting, weighted Cox regression models were used to evaluate the association between early acetaminophen exposure and mortality outcomes. Additionally, subgroup and sensitivity analyses demonstrated that the observed association remained consistent across various patient subgroups, including stratifications by inflammatory markers and comorbidities. The findings of our study suggest that acetaminophen use may be associated with improved prognosis in critically ill patients with acute ischemic stroke admitted to the ICU, and we have sought to explore the potential mechanisms underlying this observed benefit. Traditionally, acetaminophen has been utilized primarily for its antipyretic and analgesic effects in clinical practice. However, our results point to the possibility of expanding its therapeutic role in the acute management of severe ischemic stroke, potentially providing benefits that extend beyond temperature regulation. As previously discussed, the protective effects of acetaminophen may be mediated through multiple biological pathways, including modulation of neuroinflammation, attenuation of oxidative stress, improvement in cerebral perfusion, regulation of intracranial pressure, and support of post-ischemic metabolic stability. These mechanisms, acting in concert with its established antipyretic and analgesic properties, may contribute to the favorable outcomes observed in our cohort. Importantly, acetaminophen is widely available, cost-effective, and considered safe when used within therapeutic limits. These characteristics make it a clinically attractive option that could be considered for broader use in ICU patients with ischemic stroke, potentially offering a low-risk, accessible adjunctive therapy to improve outcomes in this high-risk population.

Nonetheless, several methodological considerations should be taken into account when interpreting the results. First, the retrospective nature of the study design may introduce potential biases that are inherent to observational analyses. Although inverse probability of treatment weighting was applied to adjust for observed baseline differences, the possibility of residual confounding cannot be entirely excluded. Second, due to limitations in the MIMIC-IV database, the specific indication for ICU admission could not always be confirmed, making it uncertain whether ischemic stroke was the primary reason for ICU care in all patients. Moreover, stroke severity could not be directly assessed using the National Institutes of Health Stroke Scale, which is not available in the dataset. While we used SOFA and GCS scores as proxies for physiological and neurological status, they may not fully capture stroke-specific severity. Third, as a single-center database with a distinct patient population, the MIMIC-IV dataset may limit the broader generalizability of our findings to other clinical settings or healthcare systems. Finally, although several plausible biological mechanisms were proposed—such as modulation of inflammation, oxidative stress, and cerebral perfusion—the precise pathways through which acetaminophen may exert neuroprotective effects remain to be elucidated. Future research should include prospective, multicenter randomized controlled trials to validate our findings, as well as experimental investigations at the cellular and animal levels to better understand the underlying mechanisms.

This study found that early acetaminophen use was associated with reduced mortality in ICU-admitted ischemic stroke patients. The association remained robust after adjustment using multivariable Cox regression and IPTW analysis, suggesting a potential benefit beyond fever control, possibly through modulation of inflammatory pathways. Our findings highlight acetaminophen as a readily available therapeutic option that may improve outcomes in critically ill stroke patients. However, due to the retrospective design, lack of NIHSS scores, potential residual confounding, and the single-center nature of the study, these results should be interpreted with caution. Future prospective, multicenter randomized controlled trials are necessary to validate our findings. Further mechanistic studies are also warranted to clarify how acetaminophen may confer neuroprotective effects in ischemic stroke patients.

## Data Availability

Publicly available datasets were analyzed in this study. This data can be found here: https://doi.org/10.13026/kpb9-mt58.
